# Prescription pattern of alpha-blockers for management of lower urinary tract symptoms/benign prostatic hyperplasia

**DOI:** 10.1038/s41598-018-31617-w

**Published:** 2018-09-05

**Authors:** Hyong Woo Moon, Jong Hyup Yang, Jin Bong Choi, Woong Jin Bae, Hyuk Jin Cho, Sung-Hoo Hong, Ji Youl Lee, Sae Woong Kim, Sang-Hyun Park, Kyungdo Han, U-Syn Ha

**Affiliations:** 1 Department of Urology, College of Medicine, The Catholic University of Korea, Seoul, Republic of Korea; 2 Department of Biostatistics, College of Medicine, The Catholic University of Korea, Seoul, Republic of Korea

## Abstract

This study investigated trends in the prescription of α-blockers for patients with BPH, focusing on changing patterns of prescriptions during 2002–2013 using National Health Insurance Service-National Sample Cohort data. A total of 65,596 Korean males over 50 years old diagnosed with BPH were identified from the NHIS-NSC database between 2002–2013. Patterns of each α -blocker prescription were analyzed and persistence rate, switch rate, and return rate during a follow-up period of 3 years after the first prescription were calculated. A total of 28,318 men over 50 years old (64.95 ± 9.12), changed medication within six months following the first prescription of α -blocker. (1) Tamsulosin showed the highest persistence rate when compared with other α-blockers (2) Among patients who switched to a second α-blocker, tamsulosin showed the highest return rate when compared with other α-blockers. Tamsulosin has been the most commonly prescribed α1-blocker since the mid-2000s, in line with its demonstrated highest persistence and return rates. These data probably reflect patient satisfaction with α1-blockers in the management of BPH, in which the decision to stop and switch pharmacological treatments is primarily based on changes in symptoms or side effects.

## Introduction

Lower urinary tract symptoms (LUTS) are divided into three types: storage symptoms, voiding symptoms, and post-micturition symptoms. Urinary frequency, nocturia, urgency, and incontinence are experienced during storage phase; weak stream, intermittency, hesitancy, and straining occur during voiding phase,; and post-micturition dribbling, and feeling of incomplete emptying after micturition are post-micturition symptoms. LUTS are not life-threatening; however, they can be a significant health problem, and negatively impact quality of life.

Although the causes of male LUTS are multifactorial, LUTS are thought to be results of bladder outlet obstruction (BOO) and functional change of bladder detrusor. Benign prostatic hyperplasia (BPH) as histologic diagnosis can lead to most of LUTS in men. BPH is one of the most common benign diseases in men with over 50 years old. Worldwide, the prevalence rate of BPH is more than 50% in men between 50 and 60 years of age^1^. The reported prevalence rates in Korea were between 20% and 40% in men aged 40 or older in several epidemiologic studies^2,3^.

Surgery is recommended for LUTS/BPH patients who have renal insufficiency, recurrent urinary tract infections, bladder calculi, gross hematuria, and/or LUTS refractory to medical therapy^4^. And other than absolute indication, depending on whether general anesthesia is possible and on the size of prostate, surgery method is selected among transurethral incision of the prostate(TUIP), transurethral resection of the prostate(TURP), laser vaporization, prostatectomy^5^. Before surgical intervention, pharmacologic therapy is the primary treatment for LUTS/BPH, and α_1_-adrenergic blockers are the first-choice treatment. The use of α_1_-adrenergic blockers is based on targeting both bladder α_1d_-adrenergic receptors and prostate α_1a_-adrenergic receptors, which are critical in relieving storage and obstructive symptoms^6^.

We select and prescribe various alpha-blockers in real-life clinical practice, but the study data regarding which drugs are actually prescribed and how these trends are changing over time is insufficient.

The analysis of α-blocker prescription patterns is important to assess medication adherence, adverse effects, effectiveness, and outcomes. Schneeweiss *et al*. reported that healthcare utilization databases are very useful in obtaining data about medication utilization, adverse effects, effectiveness, and outcomes^7^. Accordingly, we aimed to investigate trends in the prescription of α-blockers for patients with BPH, focusing on changing patterns of prescriptions during 2002–2013 using National Health Insurance Service-National Sample Cohort data.

## Results

From the initial cohort of 1,025,340 men, there were 65,596 were aged 50 years or older and had a BPH diagnosis. Among them, 28,318 patients aged 50 years and older with LUTS/BPH were prescribed α-blocker without anticholinergics and 5-ARI. They were divided into three groups: 4,441 who started treatment in 2002–2003, 4,112 in 2006–2007, and 5,105 patients in 2009–2010.

### General characteristics

The mean ± SD age of the study population was 64.95 ± 9.12 years. The proportion of all patients who were taking BPH-related medication increased with age, reaching a peak at 60–69 years. The majority of the patients were 60–69 years old.

### Persistence of initiating α-blocker among a population with α-blocker only medication

Table 1 shows the prescription pattern of initiating an α-blocker among the population with α-blocker only medication during the first three years of follow-up. In patients who started their prescriptions between 2002 and 2003, terazosin was the most commonly first prescribed α_1_-blocker, followed in order by doxazosin and tamsulosin. However, in patients who started their prescription in 2005–2006, the prescription of tamsulosin increased sharply, becoming the most frequent, while the proportion of terazosin and doxazosin decreased sharply. In patients who started treatment in 2009–2010, tamsulosin still was the most frequently prescribed α1-blocker, followed in order by alfuzosin, terazosin, and doxazosin.

**Table 1:** Persistence of the initial α-blocker among a population using only α-blocker medications.

Year medication/treatment started	Initiating α-blocker	Second α-blocker											
		Tamsulosin (n, %)		Alfuzosin (n, %)		Silodosin (n, %)		Doxazosin (n, %)		Terazosin (n, %)		Persistence (n, %)^†^	
2002–3	Tamsulosin	—	—	41	4.2%			188	19.2%	183	18.7%	568	58.0%
	Alfuzosin	29	20.3%	—	—			19	13.3%	26	18.2%	69	48.3%
	Silodosin					—	—					—	—
	Doxazosin	184	15.2%	61	5.0%			—	—	318	26.2%	650	53.6%
	**Terazosin**	375	17.81%	71	3.4%			383	17.5%	—	—	1350	**61**.**8%**
2006–7	**Tamsulosin**	—	—	193	10.2%			212	10.5%	196	9.7%	1263	**67**.**0%**
	Alfuzosin	88	21.1%	—	—			26	6.2%	34	8.2%	266	64.3%
	Silodosin					—	—					—	—
	Doxazosin	192	23.8%	55	6.8%			—	—	74	9.2%	480	59.9%
	Terazosin	254	22.6%	7	7.0%			120	10.7%	—	—	668	59.5%
2009–10	**Tamsulosin**	—	—	297	10.1%	153	5.5%	200	7.1%	210	7.5%	1942	**69**.**3%**
	Alfuzosin	154	22.1%	—	—	29	4.2%	57	8.2%	34	4.9%	424	60.7%
	Silodosin	66	25.5%	23	8.9%	—	—	20	7.7%	11	4.3%	139	53.7%
	Doxazosin	148	22.9%	44	6.8%	25	3.9%	—	—	59	9.1%	370	57.3%
	Terazosin	199	22.5%	49	5.5%	36	4.1%	60	6.8%	—	—	542	61.2%

Data are presented as number of patients (%: percentage of the total number of second α-blockers for each initiating α-blocker).

^†^Persistence rate: percentage of the index patients who did not change medications during the 3 years after the first prescription date of each α -blocker.

**Figure 1:**
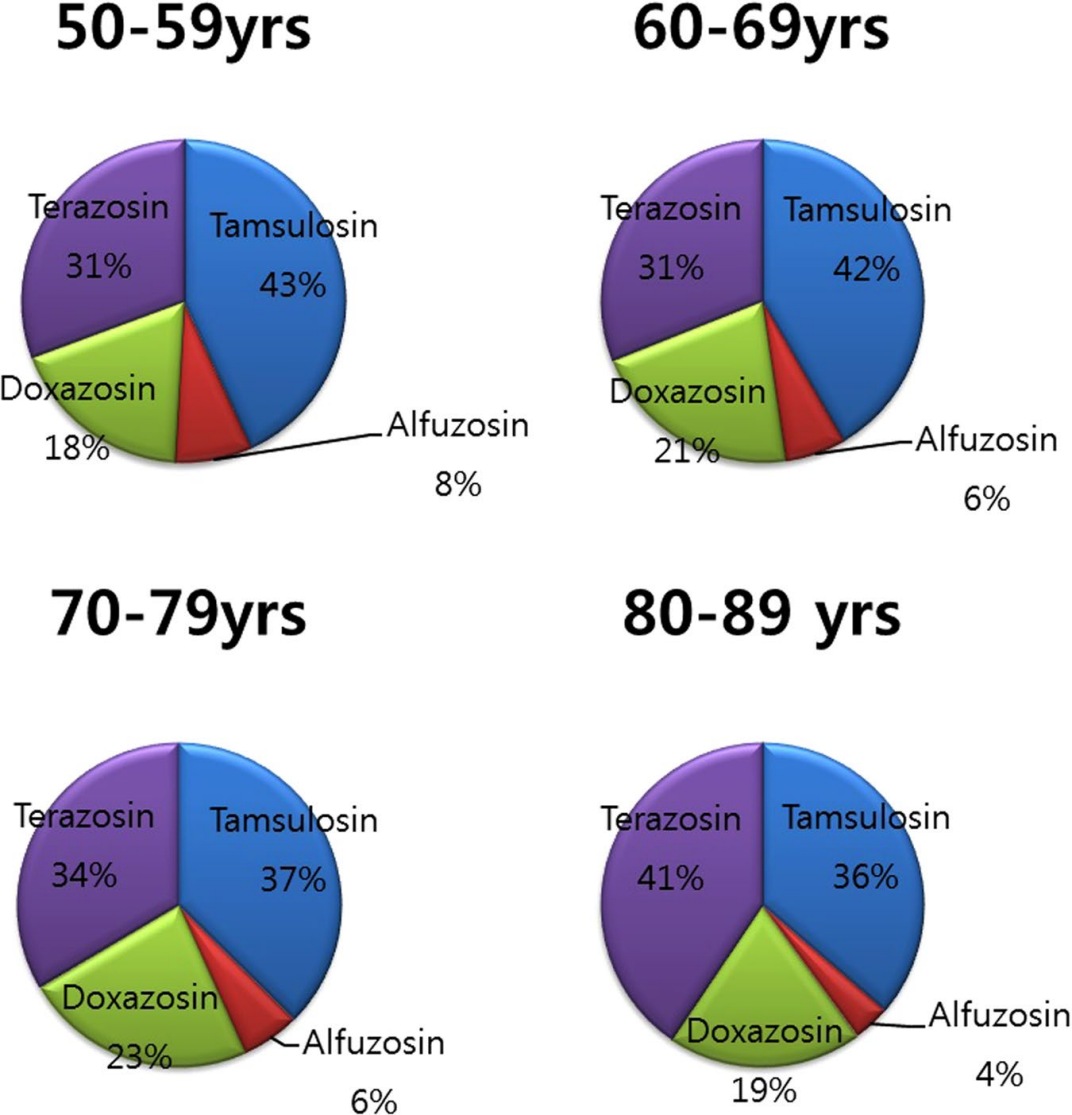
Distribution of patients who returned to BPH medication based on age group and α- blocker subtype from 2002–2010.

In contrast, regarding the persistence of initiating an α-blocker, the persistence rate varied depending on the first prescribed α1-blocker. Patients who were initiated on tamsulosin showed the highest persistence when compared with other α-blockers in the entire analysis period. This pattern (the highest persistence of tamsulosin) was seen in every age group (Fig. 1).

### Return to the initial α-blocker among the population taking a second α-blocker

Among the patients who switched to a second α-blocker, a large number were prescribed another α-blocker. Table 2 shows the pattern of return to the initial α-blocker among patients who were looking for another α-blocker following the second α-blocker during the first three years of follow-up. Patients initiated on tamsulosin showed the highest return rate when compared with other α-blockers in all analysis periods. This pattern (return to tamsulosin following second α-blocker) was seen in every age group (Fig. 1).

**Table 2:** Return to initial α-blocker among patients taking a second α-blocker.

Year medication/treatment started	Initiating α-blocker	Third α-blocker											
		Tamsulosin (n, %)		Alfuzosin (n, %)		Silodosin (n, %)		Doxazosin (n, %)		Terazosin (n, %)		Return rate^‡^ (n, %)	
2002–3	**Tamsulosin**	—	—	40	9.7%			131	31.8%	96	23.3%	145	**35**.**2%**
	Alfuzosin	20	27.0%	—	—			13	17.6%	24	32.4%	17	23.0%
	Silodosin					—	—					—	—
	Doxazosin	157	27.9%	49	8.7%			—	—	200	35.5%	157	27.9%
	Terazosin	311	37.9%	63	7.7%			220	26.8%	—	—	227	27.6%
2005–6	**Tamsulosin**	—	—	145	23.7%			128	20.9%	117	19.1%	223	**36**.**4%**
	Alfuzosin	82	52.9%	—	—			26	16.8%	22	14.2%	25	16.1%
	Silodosin					—	—					—	—
	Doxazosin	149	45.3%	53	16.1%			—	—	54	16.4%	73	22.2%
	Terazosin	189	41.4%	62	13.6%			90	19.7%	—	—	115	25.2%
2009–10	**Tamsulosin**	—	—	199	22.2%	133	14.9%	151	16.9%	157	17.5%	255	**28**.**5%**
	Alfuzosin	127	47.0%	—	—	21	7.8%	41	15.2%	28	10.4%	53	19.6%
	Silodosin	66	51.2%	19	14.7%	—	—	17	13.2%	11	8.5%	16	12.4%
	Doxazosin	127	42.8%	46	15.5%	29	9.8%	—	—	43	14.5%	52	17.5%
	Terazosin	151	42.1%	42	11.7%	30	8.4%	54	15.0%	—	—	82	22.8%

Data are presented as number of patients (%: Percentage of the total number of third α-blockers of each initiating α-blocker).

^‡^Return rate: percentage of the index patients who changed medications and were re-prescribed the first alpha blocker after the first prescription date of each alpha blocker (e.g., Tamsulosin → other alpha blocker → Tamsulosin).

## Discussion

The main findings of this population-based study are as follows: (1) tamsulosin showed the highest persistence rate when compared with other α-blockers and (2) among patients who switched to a second α-blocker, tamsulosin showed the highest return rate following initiation of a second α-blocker when compared with other α-blockers.

BPH is a unique condition that is no longer life-threatening, despite being potentially progressive^8^. Therefore, the decision to continue the medication in LUTS/BPH patients can be influenced by the ameliorated symptoms of the patients, the sense of expectancy of improvement, or side effects of drugs. When a drug is less effective than expected, patients could stop the drug or switch to other drugs. Consequently, the patients play an important role in the decision to change or stop the medication^9^.

Safety and efficacy are the most important factors when prescribing a drug. Currently there are five subtypes of α-blockers among the prescribed α-blockers; however, direct comparisons between α-blockers are limited^6^. Due to the lack of evidence regarding the differences in efficacy and safety of α-blockers, physicians make different choices among the five α-blockers when prescribing medication for LUTS/BPH.

This study demonstrated a distinct prescription pattern, which led us to speculate as to the preference for a certain prescription. A high persistence rate can reflect favorable efficacy/tolerability. Depending on the individual cases, after the symptomatic improvement with initial treatment, patients might stop taking the drug because in their minds they are completely cured of the disease.

Thus, it is impossible to conclude the favorability or superiority of a drug based on the persistence rate in our study; however, the return rate could have greater implications than the persistence rate. Also, initiating a prescription could depend on the physician’s preference.

When physicians choose a particular α-blocker over others, there can be many factors that affect the physician’s decision; however, in the case of alpha-blockers whose efficacy has not been compared, the physician’s personal prescription experience or preference can be the major factor in his or her decisions. Alternatively, the prescription choice could be determined based on the patient’s own experience of a drug’s effects and side effects as compared to the initial drug and second drug. As previously mentioned, the decision to return to the initial drug could actually be based on a comparison of satisfaction by the patient himself.

Accordingly, the finding of high persistence and return rates for tamsulosin could be potential explanations as to why prescriptions for tamsulosin increased and became the most frequent after 2005–2006 in this study. A physician’s treatment experience for α_1_-blocker medication has a considerable influence on prescriptions for new LUTS patients. These positive experiences, including high persistence and return rates, appear to be attributed to the fact that tamsulosin has been showing increasing use over other α_1_-blockers.

The main distinctive feature of our study is that, to the best of our knowledge, this is the first population-based observational study that evaluated the stop and switch trend in prescriptions for LUTS related to BPH. Until now, there have been only a few epidemiological studies evaluating the trend in drug prescriptions^10–12^. The present study was performed to show the trend for a return to initial α_1_-blocker with a wide population-based approach, which allowed us to infer a comparison of patient satisfaction for α_1_-blockers; still, direct comparisons between α -blockers have not been done.

Another distinctive feature of this study is that it was conducted over various time points in a 10-year observation period. Prescription patterns and resource use may have been influenced by the clinical trials, guidelines, and sales strategies. The present study was not conducted at a single point, but provided an analysis based on patients who began medications at different time points during the study period. Thus, our long-term consecutive analysis could be considered reliable.

One limitation of this study was that it did not include detailed clinical information such as prostate volume, symptom score, uroflowmetry, or residual volume, which can influence the response to medication. In addition, the changes in prescriptions trends were related to clinical variations. The large sample size and consecutive analysis at different time point make the present study reliable despite this limitation.

This population-based study showed a unique prescription analysis reflecting the persistence and return rates in the treatment of LUTS related to BPH. Tamsulosin has been the most commonly prescribed α1-blocker since the mid-2000s, in line with its demonstrated highest persistence and return rates. These data probably reflect patient satisfaction with α1-blockers in the management of BPH, in which the decision to stop and switch pharmacological treatment is primarily based on changes in symptoms or side effects.

## Materials and Methods

### Data sources

The Korean National Health Insurance System (NHIS) for all citizens was initiated in 1963 in accordance with the National Health Insurance Act, and it now provides benefits for prevention, diagnosis, disease and injury treatment, rehabilitation, births, deaths, and health promotion. The NHIS maintains and stores all records of healthcare utilization and prescription^13^. In this study, we used the National Health Insurance Service-National Sample Cohort (NHIS-NSC). NHIS-NSC is a population-based cohort released by the National Health Insurance Service in South Korea. A total of 1,025,340 Koreans comprised the initial 2002 cohort; of these, 2.2% of the total eligible population in 2002 (46,605,433) were randomly sampled and followed for 11 years until 2013. A representative sample of newborns has been added annually during the follow-up period, and participants that deceased or emigrated were excluded.; 1,014,730 patients were registered on the database in 2013.

### Study design

**Figure 2:**
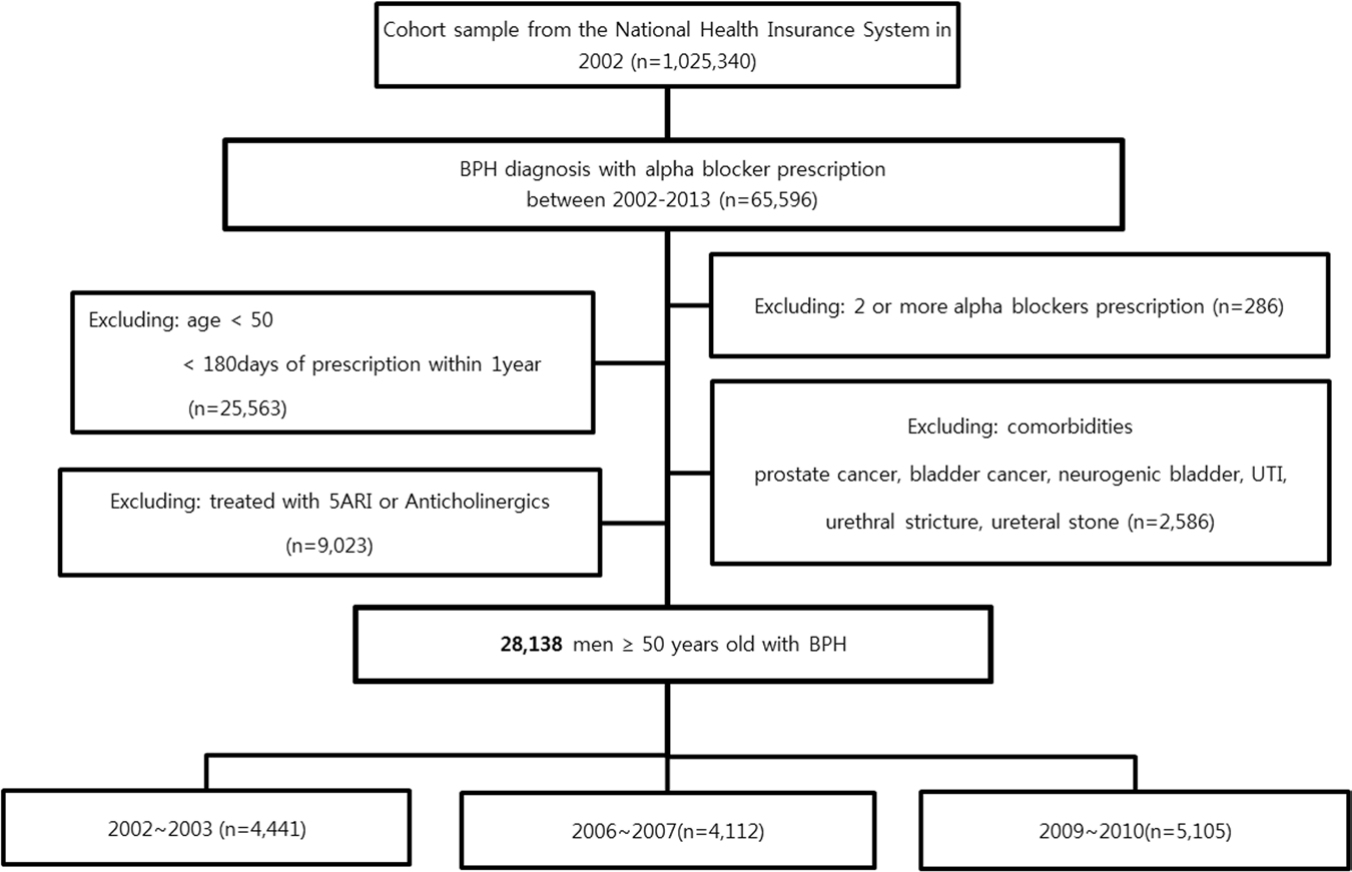
Sample selection flow chart for the index period from January 1, 2002 to December 31, 2013.

Patients with BPH were identified by searching for codes in the 10th Revision of International Classification for Diseases (ICD-10), N40. The study period includes prescriptions from January 1, 2002 to December 31, 2013, and was divided into three groups based on the year medication was initiated (2002–2003, 2006–2007, and 2009–2010; Fig. 2).

We analyzed patterns in alpha-blocker prescription for three years after the first prescription by each group. Patients were eligible for inclusion if they were 50 years or older at the time of first alpha-blocker prescription and diagnosed with BPH. Patients were also required to have over 180 days of prescription within one year of their first prescription of alpha-blocker. Patients were excluded from the study if they were prescribed another BPH medication such as 5-alpha-reductase inhibitor (5-ARI) or an anticholinergic. We also excluded patients who had comorbidities such as prostate cancer (ICD-10,C61), bladder cancer (C679), neurogenic bladder (N319), urinary tract infection (N390), urethral stricture (N350, N358, N359), ureteral stone (N201, N202), or BPH-related procedure, which included transurethral surgery (TUR-P, laser vaporization, Urolift) and simple prostatectomy. This study was approved by the Institutional Review Board of The Catholic University of Korea (KC16EISI0583). All information used for statistical analysis was anonymized, and informed consent was waived. All research methods were performed in accordance with the relevant guidelines and regulations.

### Definition of the prescription pattern

The patterns of α-blocker prescription were analyzed, and the persistence rate and return rate during a follow-up period of 3 years after the first prescription were calculated. Persistence rate was defined as the percentage of the index patients who had not changed medication during the three years after the first date of prescription of an α-blocker. Cases of changes after three years from the first prescription are regarded as ‘no change’. This is to keep the same length of observation period on a yearly basis. If a patient was prescribed a second α-blocker included in the study within the 3-year follow-up, the situation was defined as a ‘switch.’ If a patient had a second switch and the third medication was the same as the first medication included in the study within the 3-year follow-up, it was defined as a ‘return’. We have divided the patients whose age range from 50s–80s by the category of the 50 s, 60 s, 70 s, and 80 s, and reported the return rate for each category. The time periods in which the five drugs were prescribed were 2002–2003, 2006–2007, and 2009–2010, and we have examined the prescription records for each patient who have first received alpha-blockers for three years. This was done to keep the length of observation period the same across the board. Data were analyzed using descriptive statistics, and results are shown as rates.
